# Improvement of In Vitro Seed Germination and Shoot Development of the Indonesian Endangered Orchid, *Dendrobium lineale* Rolfe, Using Sucrose and Coconut Water

**DOI:** 10.1155/sci5/2153196

**Published:** 2026-01-02

**Authors:** Edy Setiti Wida Utami, Sri Lestari, Dwi Kusuma Wahyuni, Junairiah, Hery Purnobasuki, Sucipto Hariyanto, Nabila Istighfari, Ahmad Affan Ali Murtadlo, Siti Umamah

**Affiliations:** ^1^ Department of Biology, Faculty of Science and Technology, Universitas Airlangga, Surabaya, Indonesia, unair.ac.id; ^2^ Biodivesity and Biotechnology of Tropical Plant Research Group, Universitas Airlangga, Surabaya, Indonesia, unair.ac.id; ^3^ Plant Structure and Development Laboratory, Faculty of Biology, Jenderal Soedirman University, Purwokerto, Indonesia, unsoed.ac.id; ^4^ Advance Tropical Genomic and Conservation Research Group, Universitas Airlangga, Surabaya, Indonesia, unair.ac.id; ^5^ Magister Science Biology Program, Faculty of Science and Technology, Universitas Airlangga, Surabaya, Indonesia, unair.ac.id; ^6^ Biology Undergraduate Study Program, Department of Biology, Faculty of Science and Technology, Universitas Airlangga, Surabaya, Indonesia, unair.ac.id

**Keywords:** coconut water, endangered orchid, in vitro propagation, sucrose

## Abstract

*Dendrobium lineale* Rolfe is an important orchid species used as a parent in breeding. Currently, this orchid is classified as an endangered species and is listed in Appendix II according to CITES. This study aimed to investigate the effects of sucrose and coconut water (CW) on the asymbiotic seed germination and shoot development of this endangered Indonesian orchid. *In vitro* orchid seed culture aids in conservation and reintroduction. Three‐month‐old hand‐pollinated seeds were sown on Vacin and Went (VW) solid medium supplemented with several concentrations of sucrose (10, 20, and 30 g/L) or without sucrose (controls). Seed germination and protocorm development were investigated 1, 2, and 3 months after sowing. To analyze the role of CW in subsequent shoot development and root formation, shoots with an approximately 1‐cm length and comprising 1‐2 leaves obtained following seed germination were cultured on VW medium supplemented with several concentrations of CW (5%, 10%, 15%, and 20%) and without CW (control) treatment. After 4 months of culture, the number of roots, the number of leaves, and dry weight of the plantlets were recorded. VW medium containing 20 g/L sucrose effectively enhanced seed germination (89%) and shoot formation with one or more leaves (stage 5) (46%). Supplementation with 20% CW in VW medium was suitable for shoot development, resulting in well‐developed roots and leaves and improved fresh weight of plantlets compared with those in the other treatments. Moreover, 87% of the acclimatized plantlets survived. This protocol is an efficient method for the in vitro mass production and conservation of this endangered epiphytic orchid using asymbiotic seed culture.

## 1. Introduction

Orchidaceae, the second‐largest plant family, has approximately 29,524 species [1–3]. However, an estimated 25% of all orchid species are at imminent risk of extinction owing to many factors, including overexploitation, illegal trade, and habitat loss. Therefore, conservation is a solution for maintaining orchid species, with plant tissue culture techniques being effective conservation methods [4–6].

*Dendrobium lineale* Rolfe is an orchid with great potential as a cut flower and is a parent of orchid hybrids. *D. lineale* is endemic to Papua Island in Indonesia. Currently, the existence of this species and its natural habitat is threatened by extinction due to habitat destruction and overexploitation. In nature, *D. lineale* is an endangered species and is listed in Appendix II according to the Convention on International Trade in Endangered Species of Wild Fauna and Flora (CITES). *D. lineale* is not currently classified as a threatened species; nevertheless, international trade is regulated and permitted only with the issuance of valid export permits in accordance with CITES provisions [7]. Micropropagation of orchids through in vitro seed culture can facilitate the production of large members of orchids in a relatively short time. Asymbiotic orchid seed germination is influenced by several factors, including the type of culture media [8–10], pod maturity [11–13], and plant growth regulators [14, 15]. Adding sucrose to culture media has also been shown to effectively enhance the seed germination and protocorm development in *Cypripedium macranthos* [16]. Organic compound supplements also affect shoot development, seedling growth, and in vitro regeneration [17, 18]. These organic compounds include peptone (P), potato homogenate (PH), banana homogenate (BH), tomato juice (TJ), chitosan (CHT), and coconut water (CW) [19, 20]. De Stefano et al. [21] reported that culture media with organic supplements resulted in greater plant length and number of roots than those in the *Epidendrum nocturnum* control. In the present study, we evaluated the effect of various concentrations of sucrose (0, 10, 20, and 30 g/L) and CW (0%, 5%, 10%, 15%, and 20%) on seed germination and shoot development in *D*. *lineale*. We established a reproducible protocol for rapidly and efficiently propagating *D*. *lineale* through in vitro seed germination. The results of this study will be useful for the conservation of this species.

## 2. Materials and Methods

### 2.1. Capsule Collection and Sterilization

Three‐month‐old mature capsules, derived from hand pollination, were collected from the DD Orchid Nursery, East Java, Indonesia. To eliminate dust, capsules were washed with 10% detergent solution for 10 min and then rinsed with sterile water thrice. This process was followed by sterilization with 5% sodium hypochlorite solution for 10 min. Thereafter, the samples were than rinsed thrice with sterile water and placed in a sterile Petri dish in a laminar flow hood. Next, the capsules were dissected longitudinally into two halves using a sterile surgical blade and the seeds were pooled.

### 2.2. Seed Morphological Observation

An average of 30 seeds was observed and photographed under a tension light stereomicroscope (Nikon NMZ; Nikon, Tokyo, Japan). Qualitative data on general seed morphology included seed shape, seed color, embryo shape, and embryo color.

### 2.3. Histology Analysis

For histological observations, microscopic slides were prepared using the paraffin method. The seeds were fixed in FAA (70% ethyl alcohol:glacial acetic acid:formaldehyde at 90:5: 5 v/v/v), dehydrated in ethyl alcohol series, and then embedded in paraffin wax for 24 h. Afterward, longitudinal sections of 8 μm thickness were prepared using a rotary microtome (Shibuya, Japan). The sections were stained with 1.5% safranin and 1.0% fast green and then mounted with Canada Balsam Synthetic in xylene (Aldon Corporation, Avon, NY, USA). Seed histology was observed using an Olympus FSX100 light microscope (Olympus, Tokyo, Japan). Seed and embryo sizes (length and width) were measured (at the longest and widest axes) using a light microscope with a micrometer. Data on length and width were collected from 30 replicates.

### 2.4. Influence of Sucrose on Seed Germination and Protocorm Development

To evaluate the influence of sucrose on seed germination and protocorm development, seeds were sown on Vacin and Went (VW) medium [22] supplemented with 10, 20, or 30 g/L sucrose (Merck, Darmstadt, Germany), and the medium without sucrose was used as a control. All media were solidified with 2 g/L gellan gum (Phytagel, Sigma‐Aldrich, St. Louis, MO, USA). The pH of the media was adjusted to 5.7 using 0.1M HCl or KOH before the addition of gellan gum. The medium was autoclaved at 120° C for 15 min. For each treatment, approximately 200 seeds were cultured in bottles containing medium. All treatments were repeated for thrice with five culture bottles for each repetition. After 1, 2, and 3 months of culture, seed germination and embryonic development were observed using an SM stereomicroscope (Nikon). The process of seed germination until the protocorm developed into the shoot was classified into five stages according to the stages of embryonic development: Stage 0: seed, Stage 1: swollen embryo, still covered by testa, Stage 2: ruptured testa = germination, Stage 3: embryo detaches from the testa = protocorm, Stage 4: protocorm with apex shoot differentiation, and Stage 5: protocorm with first, second, or third leaves = shoots. The percentage at any stage of embryo development was calculated by dividing the number of embryos at each stage by the total number of seeds observed x 100 (including viable and nonviable seeds).

### 2.5. Evaluation of CW Effect on *D. lineale* Shoot Development

After 3 months of culture, shoots obtained from seeds germination were subcultured individually in new VW medium. Shoots with a length of 6–10 mm and 1‐2 leaves were grown on VW medium supplemented with various CW concentrations (5%, 10%, 15%, and 20%), with medium without CW supplementation used as the control treatment. Each treatment was replicated thrice in five culture bottles containing five shoots. All cultures were maintained under a 16‐h light and 8‐h darkness cycle at 22 ± 2° C. After 4 months of culture, the plantlets were released from the bottles and washed with running water to remove the agar. The number of roots and leaves and the fresh weight of the plantlets were recorded.

### 2.6. Experimental Design and Data Analysis

The experimental units were set up using a completely randomized design. Data were analyzed using ANOVA in SPSS (Version 22; SPSS Inc., Chicago, IL, USA). Mean values were separated using Duncan’s multiple range test at a level of significance of *α* = 0.05 [23].

## 3. Results

### 3.1. Morphological and Anatomical Observation of Seeds

Mature *D. lineale* seeds, which were used as explants in this study, were microscopic in size (546 μm in length and 120 μm in width). The seeds were transparent and fusiform in shape (Figure 1(a)). The cellular organization of the seeds was also simple, comprising only an undifferentiated mass of embryonic cells, with no protection of the endosperm from the testa (Figure 1(b)). Similar to the seeds, *D. lineale* embryos were also simple, with an oval embryo shape, light orange color, and located at the center of the seed (450 μm in length and 85 μm in width). In the present study, *D. lineale* had a large embryo that occupied considerable space in its seed (Figure 1(b)).

Figure 1Embryo development of *Dendrobium lineale* in vitro. (a, b) Stage 0: mature seed with intact testa and an oval embryo shape, (c) Stage 1: swollen embryo, (d) Stage 2: ruptured testa (germination), (e) Stage 3: embryo detaches from the testa (protocorm), (f) Stage 4: protocorm with shoot apical differentiation, (g) Stage 5: protocorm with the first, second, or third leaves, and (h) seedling with two or more expanded leaves and developed roots. e = embryo, t = testa, sd = shoot apical differentiation, and r = rhizoid.

**Figure:**
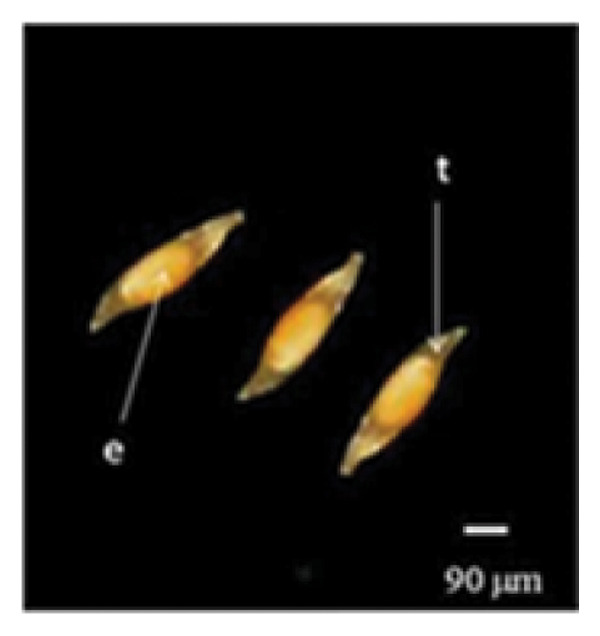
(a)

**Figure:**
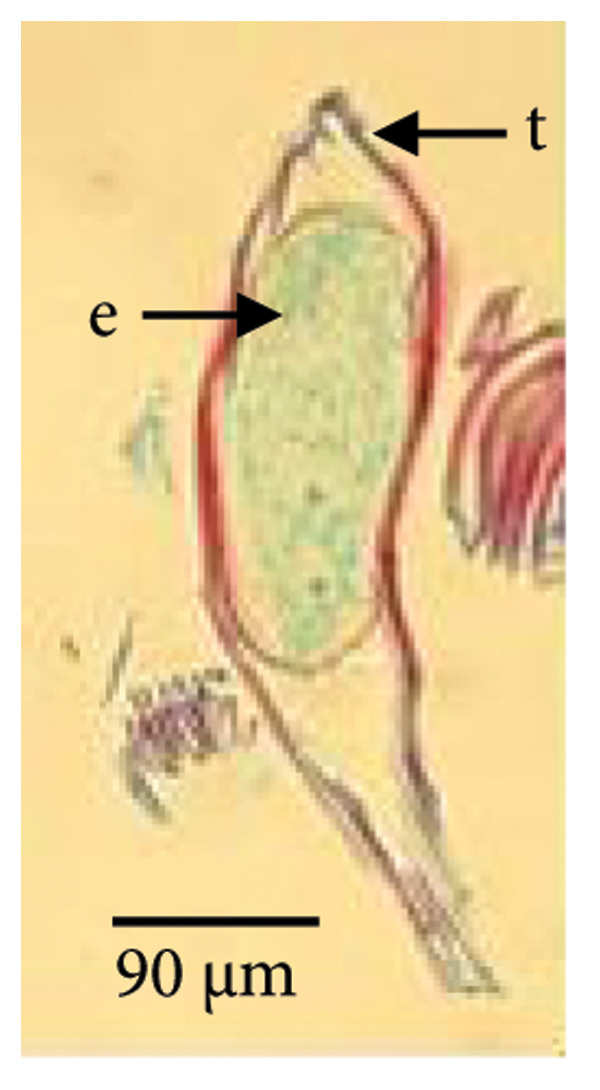
(b)

**Figure:**
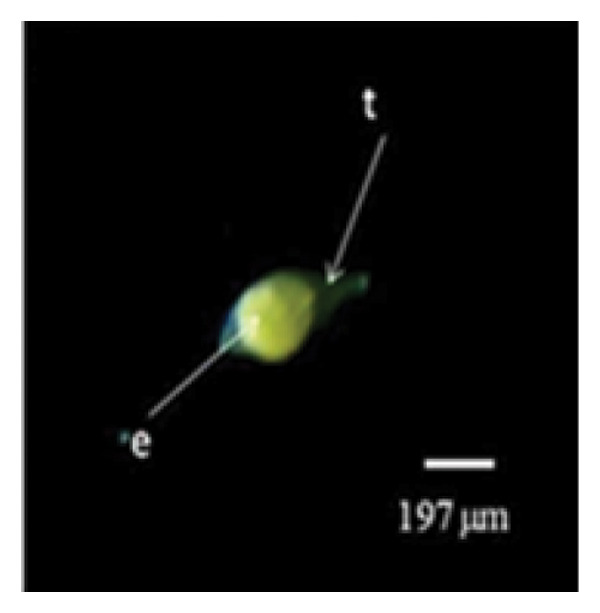
(c)

**Figure:**
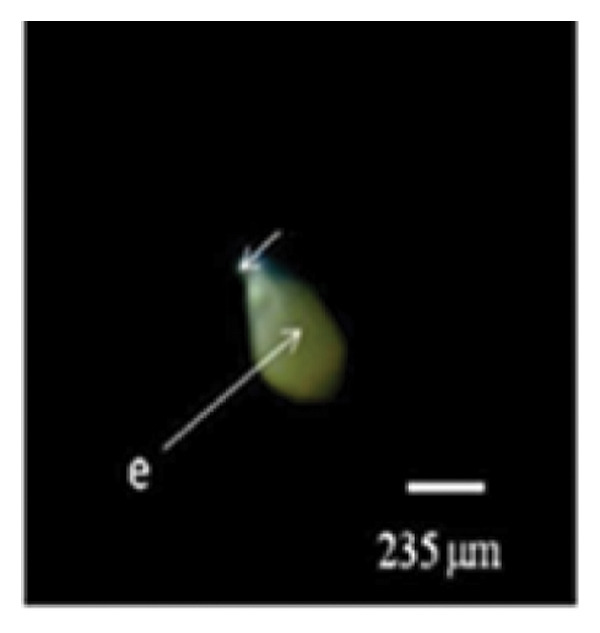
(d)

**Figure:**
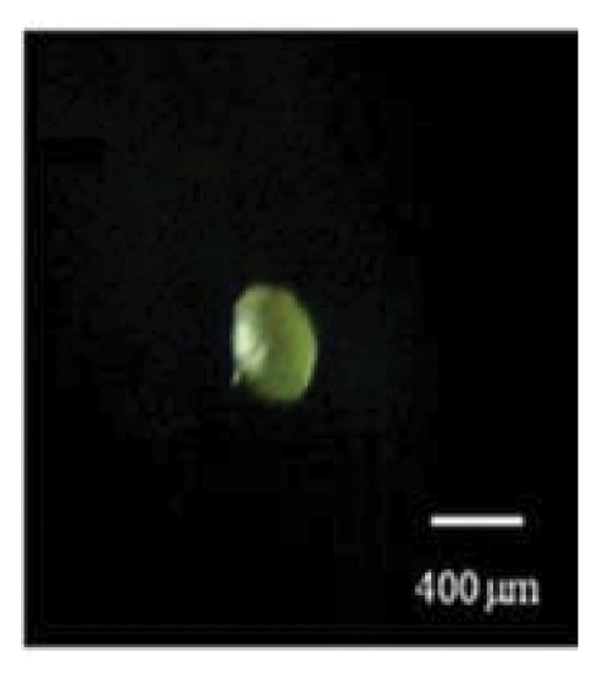
(e)

**Figure:**
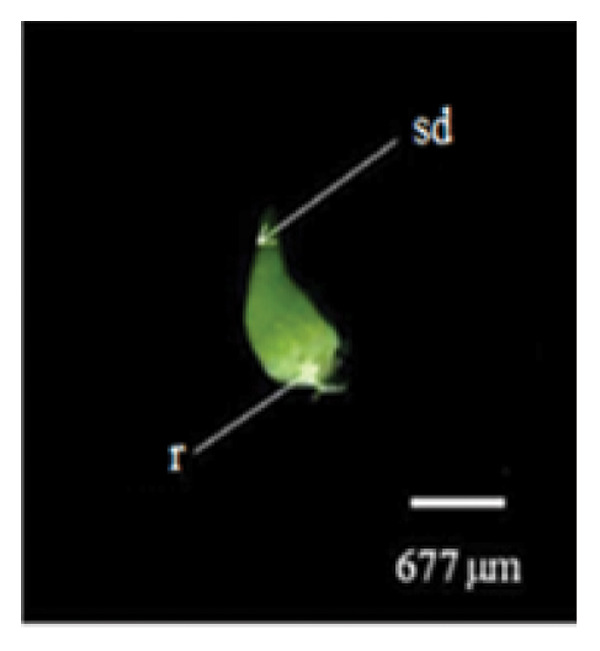
(f)

**Figure:**
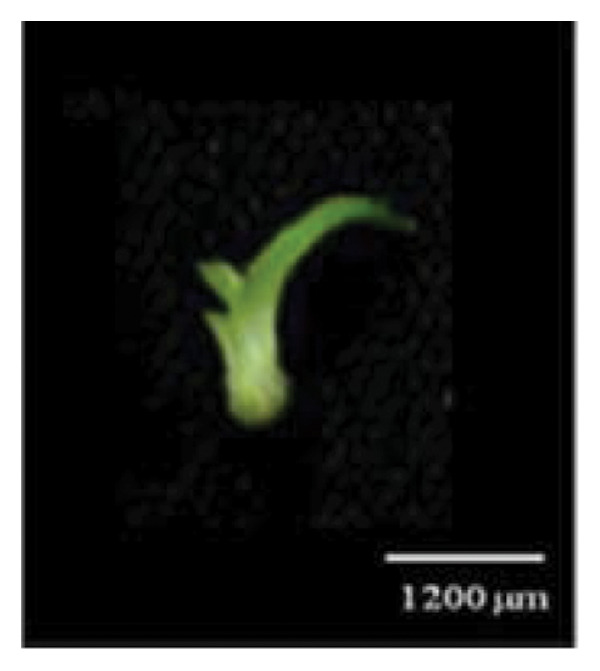
(g)

**Figure:**
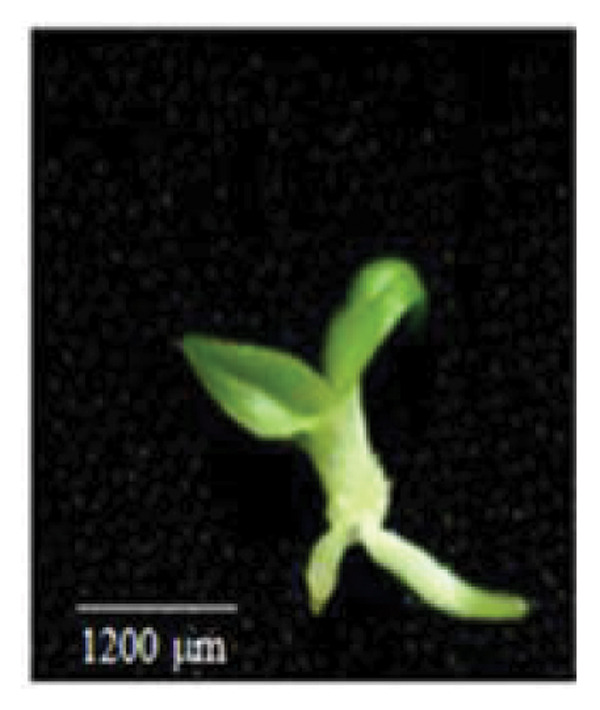
(h)

### 3.2. Seed Germination and Protocorm Development In Vitro

Figure 1 illustrates the morphological developmental stages of *D. lineale* from embryo to plantlet. The first sign of in vitro seed germination was embryo swelling, green (Figure 1(c)), approximately 2 weeks after culture, followed by testa rupture, which occurred 3 weeks after culture (Figure 1(d)). The embryo grew larger, emerged from the testa, and developed into a round, light green protocorm (Figure 1(e)). Upon reaching approximately 677 µm, the protocorm exhibited elongation, with rhizoid formation and shoot apical differentiation (Figure 1(f)), subsequently followed by the emergence of the first and second leaves (Figure 1(g)). Finally, the roots appeared when shoots were subcultured in VW medium supplemented with sucrose (Figure 1(h)).

### 3.3. Sucrose Effects on Seed Germination and Protocorm Development

The effects of sucrose on seed germination and protocorm development in *D. lineale* were evaluated at 1–3 months (Tables 1, 2, 3). After 1 month of culture (Table 1), the percentage of minimum seed germination in VW medium with 0, 10, 20, and 30 g/L sucrose was 5.6%, 46.0%, 76.6%, and 86.7%, respectively. Shoots (Stage 5) were not formed in any treatments. After 2 months of culture (Table 2), shoot formation (Stage 5) occurred only in the medium with 20 g/L (12%) and 30 g/L sucrose (35%). The seed germination rate in the treatment without sucrose (6.4%) was lower than that in VW medium containing 10 g/L (59.6%), 20 g/L (79.8%), and 30 g/L sucrose (87.0%). After 3 months of culture (Table 3), the seed germination rate in the medium without sucrose was the lowest (20.7%) compared to media added 10 g/L (62.0%), 20 g/L (89.0%), and 30 g/L (88.3%) of sucrose. Stage 5 was observed in media with 10 g/L (14.0%), 20 g/L (46.0%), and 30 g/L sucrose (33.7%). Despite germination, the embryos of this species were unable to develop into shoots (Stage 5) when cultured in media without sucrose.

**Table 1:** Effects of sucrose on seed germination and protocorm development of *Dendrobium lineale* at 1 month after culture.

Sucrose concentration (g/L)	Stages of embryo development of *D. lineale* Rolfe (%)						Total germination (%)
	0	1	2	3	4	5	
0	55.7 ± 1.6^a^	38.7 ± 3.2^a^	5.6 ± 1.6^b^	0^d^	0^c^	0	5.6 ± 1.6^d^
10	28.7 ± 1.7^b^	25.3 ± 1.7^b^	11.3 ± 1.7^a^	34.7 ± 1.7^c^	0^c^	0	46.0 ± 3.4^c^
20	17.7 ± 1.6^c^	5.7 ± 1.6^c^	3.0 ± 1.0^c^	61.0 ± 1.6^a^	12.6 ± 1.4^b^	0	76.6 ± 1.4^b^
30	10.7 ± 1.4^d^	2.6 ± 1.4^d^	1.9 ± 1.7^c^	42.9 ± 1.0^b^	41.9 ± 1.7^a^	0	86.7 ± 1.0^a^

*Note:* Means ± SD in each stage followed by the same letter indicate no significant difference based on Duncan’s multiple range test at *p* = 0.05.

**Table 2:** Effects of sucrose on seed germination and protocorm development of *Dendrobium lineale* at 2 months after culture.

Sucrose concentration (g/L)	Stages of embryo development of *D. lineale* Rolfe (%)						Total germination (%)
	0	1	2	3	4	5	
0	55.0 ± 1.7^a^	38.6 ± 2.4^a^	6.4 ± 1.0^a^	0^d^	0^c^	0^c^	6.4 ± 1.0^d^
10	25.7 ± 1.6^b^	14.7 ± 1.7^b^	8.7 ± 1.7^a^	39.6 ± 1.0^b^	11.3 ± 1.7^b^	0^c^	59.6 ± 2.9^c^
20	15.6 ± 1.6^c^	4.6 ± 1.7^c^	7.6 ± 1.6^a^	47.6 ± 1.6^a^	12.6 ± 1.4^b^	12.0 ± 1^b^	79.8 ± 3.1^b^
30	10.3 ± 1.0^d^	2.6 ± 1.4^c^	0^b^	34.7 ± 1.7^c^	17.3 ± 1.4^a^	35.0 ± 1^a^	87.0 ± 1.0^a^

*Note:* Means ± SD in each stage followed by the same letter indicate no significant difference based on Duncan’s multiple range test at *p* = 0.05.

**Table 3:** Effects of sucrose on seed germination and protocorm development of *Dendrobium lineale* at 3 months after culture.

Sucrose concentration (g/L)	Stages of embryo development of *D. lineale* Rolfe (%)						Total germination (%)
	0	1	2	3	4	5	
0	50.7 ± 1.4^a^	28.3 ± 1.7^a^	20.7 ± 1.4^a^	0^c^	0^c^	0^d^	20.7 ± 1.4^c^
10	24.0 ± 1.4^b^	14.0 ± 1.4^b^	0^b^	25.3 ± 1.7^b^	22.7 ± 1.4^a^	14.0 ± 1.4^c^	62.0 ± 2.8^b^
20	11.0 ± 1.6^c^	0^c^	0^b^	31.0 ± 1.6^a^	12.0 ± 1.7^b^	46.0 ± 2.1^a^	89.0 ± 1.6^a^
30	11.7 ± 1.7^c^	0^c^	0^b^	32.3 ± 1.6^a^	22.3 ± 1.6^a^	33.7 ± 2.4^b^	88.3 ± 1.7^a^

*Note:* Means ± SD in each stage followed by the same letter indicate no significant difference based on Duncan’s multiple range test at *p* = 0.05.

### 3.4. The Effect of CW on Subsequent Shoot Development

The effect of CW on subsequent shoot development of *D. lineale* was evaluated at 4 months (Figure 2). CW was also added to increase shoot development and root formation. In this study, the presence of CW in VW medium resulted in a better response than that without CW (control treatment). The maximum response was obtained from VW medium supplemented with 20% CW. Here, 100% of the samples responded with an average number of 6.5 roots/shoot and 6.3 leaves/shoot, significantly differing from other treatments. The highest fresh weight of plantlets (0.38 g) was observed when shoots grew in this medium, also significantly different from that of the other treatments.

Figure 2Effects of various CW concentrations on subsequent shoot development of *Dendrobium lineale* at 4 months of culture. (a) Number of roots, (b) number of leaves, and (c) fresh weight of plantlets. Data are presented as means ± SD. The same letter indicates no significant difference based on Duncan’s multiple range test at *p* = 0.05.

**Figure:**
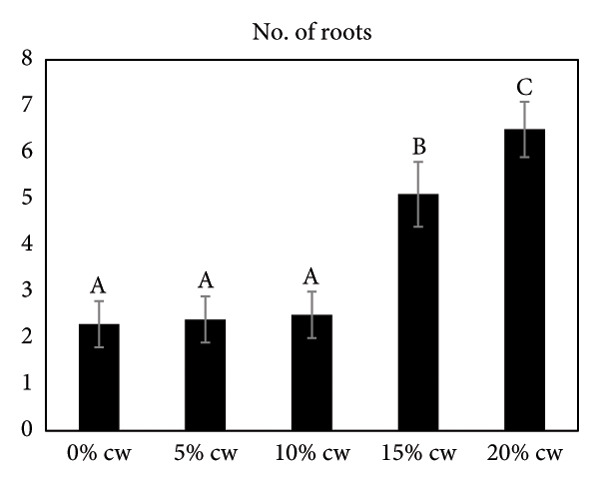
(a)

**Figure:**
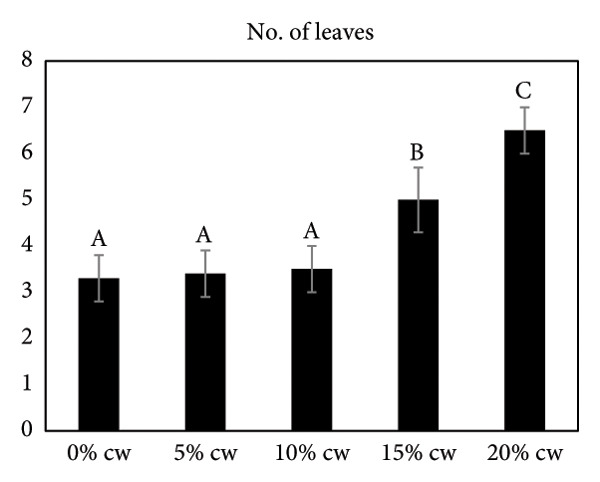
(b)

**Figure:**
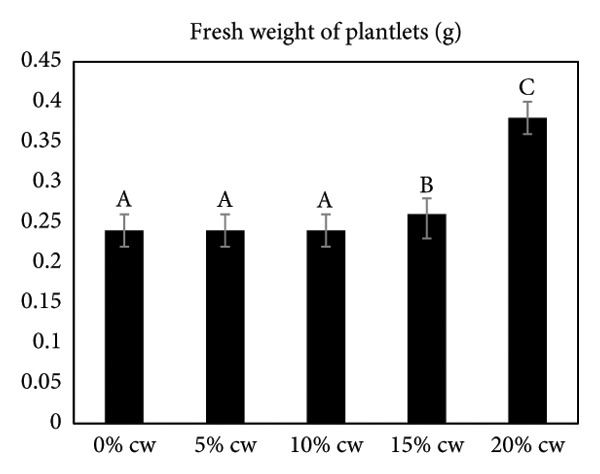
(c)

## 4. Discussion

Orchid seeds have limited carbohydrate storage, which is needed for germination and protocorm development. Therefore, an exogenous carbohydrate source in the form of sugar is required for in vitro germination [24]. Soluble sugars in *in vitro* media are usually added in the form of glucose, fructose, and sucrose in concentrations between 10 and 30 g/L [25, 26]. The addition of sucrose as the carbon source in culture media has been demonstrated to be very important in *in vitro* seed germination and protocorm development [27, 28]. Therefore, we investigated the role of sucrose on seed germination and protocorm development of *D. lineale*. The addition of 10–30 g/L sucrose to VW medium significantly enhanced the rate of *D. lineale* seed germination (Tables 1, 2, and 3). Stage 5 protocorms with first, second, or third leaves were present only when the seeds were cultured in media containing sucrose. Our results indicated that *D. lineale* seeds require sucrose for germination and embryo development in vitro. The results of this study were supported by Diantina et al. [29], who reported that Murashige and Skoog (MS), ½ MS, Norstog, and VW media containing sucrose supported seed germination in *Dendrobium strebloceras* better than media without sucrose and also induced better protocorm development with the largest protocorms recorded with the addition of 20 g/L sucrose. Ragu et al. [30] also found that ½ MS medium supplemented with 10 g/L sucrose was more effective at enhancing the seed germination of *Paphiopedilum lowii*. Longchar and Deb [28] demonstrated that the optimum seed germination of *Dendrobium heterocarpum* (95%) was achieved on nutrient medium containing sucrose than fructose (81.5%) and glucose (49.9%). According to Mercado and Delgado [31], the positive effect of sucrose is correlated with the activation of seed metabolism and aids in preserving osmotic stability between the seed and the environment, controlling solution absorption, and minimizing embryo blemishing.

Organic compound supplements increase plant tissue development, especially in *in vitro* orchid cultures. Organic compounds include PH, BH, P, TJ, yeast extract, CHT, apple homogenate, corn extract, pineapple juice, casein hydrolysate, and CW [32–36]. Organic nutrients are supplemented into culture medium as natural vitamin sources, carbohydrates, amino acids, peptides, fatty acids, and growth factors, all of which facilitate orchid growth and development. In the present study, we assayed the effects of CW on *D. lineale* root induction and shoot development. The shoots grown in VW media with 20% CW showed significantly better performance for all analyzed variables compared with those grown without CW or in medium supplemented with 5%, 10%, and 15% CW (Figure 2). Our results indicate that *D. lineale* shoots require high CW concentration for subsequent shoot development and root formation. The beneficial influence of CW on root formation and shoot development may be related to the fact that CW contains auxins, cytokinins, and gibberellins, which are critical plant hormones involved in root formation, cell division, and overall growth regulation [37, 38]. Auxin, a plant growth regulator, is one of the most important and useful components of CW in plant tissue culture. Auxins are plant growth regulators that play important roles in plant development including differentiation, root formation, gametogenesis, and embryogenesis. Auxins play essential roles in growth, including cell division and seedling growth, and stimulate plant cells to elongate [39, 40]. Aung et al. [41] reported that the addition of CW 150 mL/L to MS medium significantly affected callus induction and the development of protocorm‐like bodies in the endangered orchid *Bulbophyllum auricomum* Lindl. Fatahi et al. [42] found that Malmgren medium supplemented with CW 100 mL/L was the best medium for protocorm and plantlet growth of the endangered terrestrial orchid species *Orchis simia*. Pant et al. [43] also found that FMS medium supplemented with 10% and 5% CW speeds up initiation of seed germination and protocorm formation in *Dendrobium chryseum* Rolfe better than FMS medium containing phytohormones 6‐benzylaminopurine (BAP) and 1‐naphthaleneacetic acid (NAA). The highest number of shoots from the protocorm of *Dendrobium densiflorum* Lindl was reported by Pant et al. [44] in full‐strength MS medium containing CW 150 mL/L. Three months after transplantation, plantlets were successfully acclimatized to greenhouse conditions, with a survival rate of more than 87% (Figure 3).

Figure 3Shoot development and establishment of *Dendrobium lineale* Rolfe plantlets. (a) Development of healthy shoots of *D. lineale* after 4 months of culture on VW medium supplemented with 20% CW. (b) Transplanted plantlets in the mixture of coconut fiber and sphagnum moss (3:1 v/v) following Utami et al. [45] after 3 months of acclimatization in a greenhouse. Scale bars: (a) 10 mm; (b) 15 mm.

**Figure:**
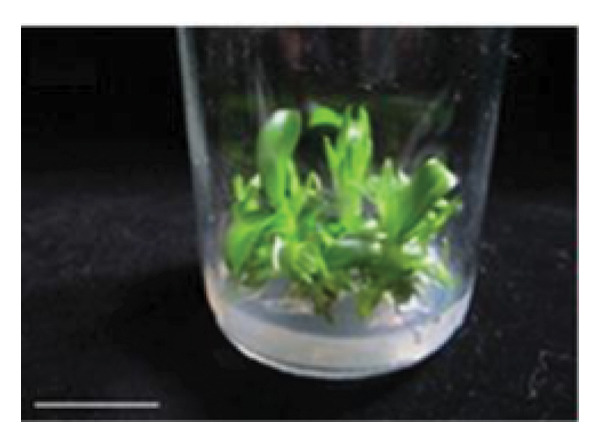
(a)

**Figure:**
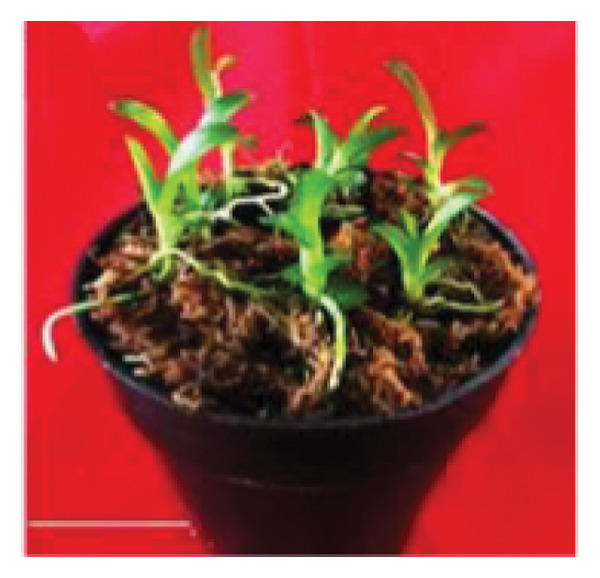
(b)

## 5. Conclusions

In this study, we investigated the effect of sucrose and CW on the asymbiotic seed germination of *D. lineale*, an endangered Indonesian ornamental orchid. We found that sucrose significantly affected *D. lineale* seed germination. The optimal sucrose concentration for asymbiotic germination and protocorm development was 20 g/L. The addition of CW to culture medium increased shoot development. The highest number of leaves, root formation, and fresh weight of plantlets was observed when shoots were grown in the presence of 20% CW. Therefore, an efficient method was developed for the germination and plantlet regeneration of mature seeds to establish *D. lineale*.

## Conflicts of Interest

The authors declare no conflicts of interest.

## Funding

This research received no external funding.

## Supporting Information

The supporting material consists of the brochure *“Index of CITES Species”* produced by the CITES Secretariat in collaboration with UNEP‐WCMC. This document provides an authoritative summary of taxa listed in the CITES Appendices, including standardized scientific nomenclature, regulatory annotations, and relevant distribution information. The brochure outlines the structure and function of the CITES Species Checklist and serves as an essential reference for ensuring consistent taxonomy and compliance with international trade regulations under CITES.

## Supporting information


**Supporting Information** Additional supporting information can be found online in the Supporting Information section.

## Data Availability

The data that support the findings of this study are available from the corresponding author upon reasonable request.
