# Supplementary material for: Improvement of In Vitro Seed Germination and Shoot Development of the Indonesian Endangered Orchid, Dendrobium lineale Rolfe, Using Sucrose and Coconut Water
Source: Scientifica (Cairo). 2026 Jan 2;2026:2153196. doi: 10.1155/sci5/2153196 (PMC12782341; doi:10.1155/sci5/2153196)
Supplement: Supplementary file 1 — Supporting Information Additional supporting information can be found online in the Supporting Information section. [file SCI5-2026-2153196-s001.pdf]

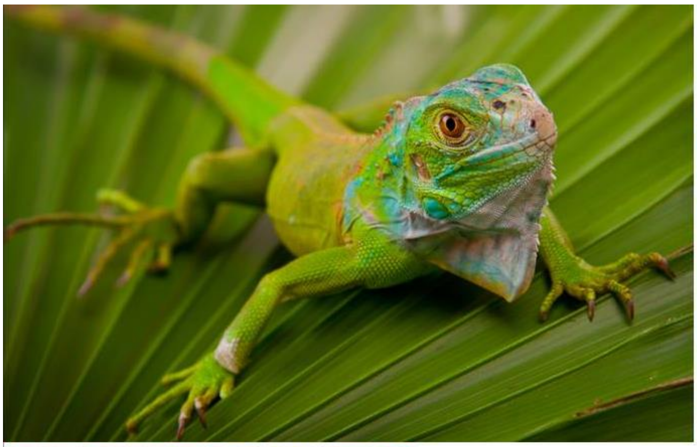
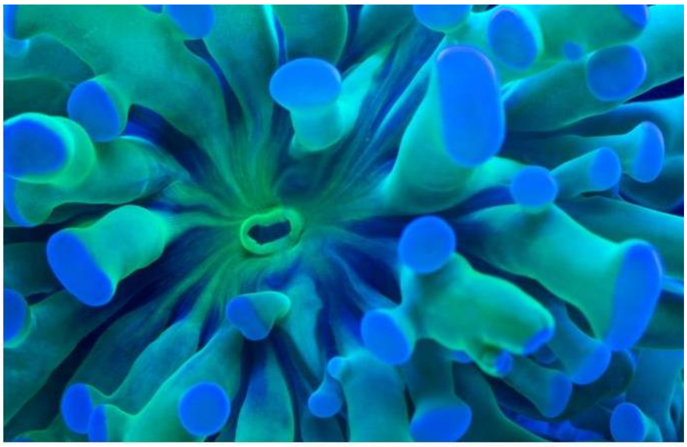
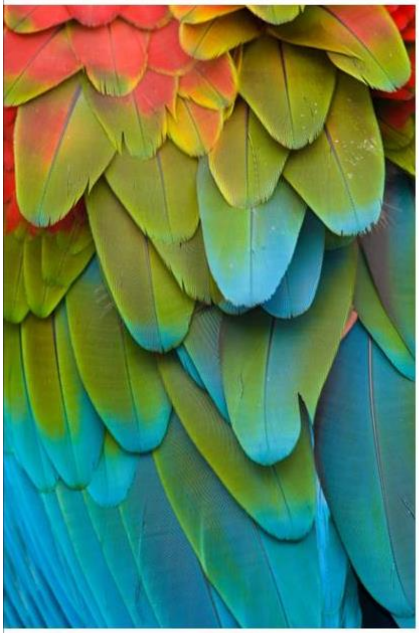

Index of CITES species

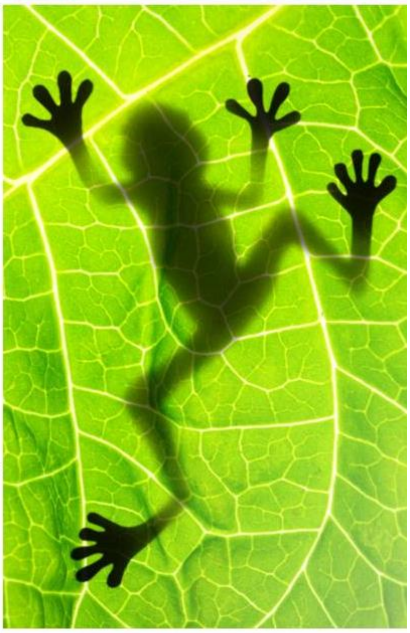
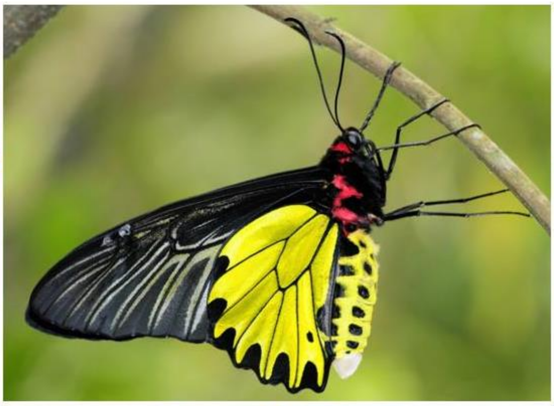
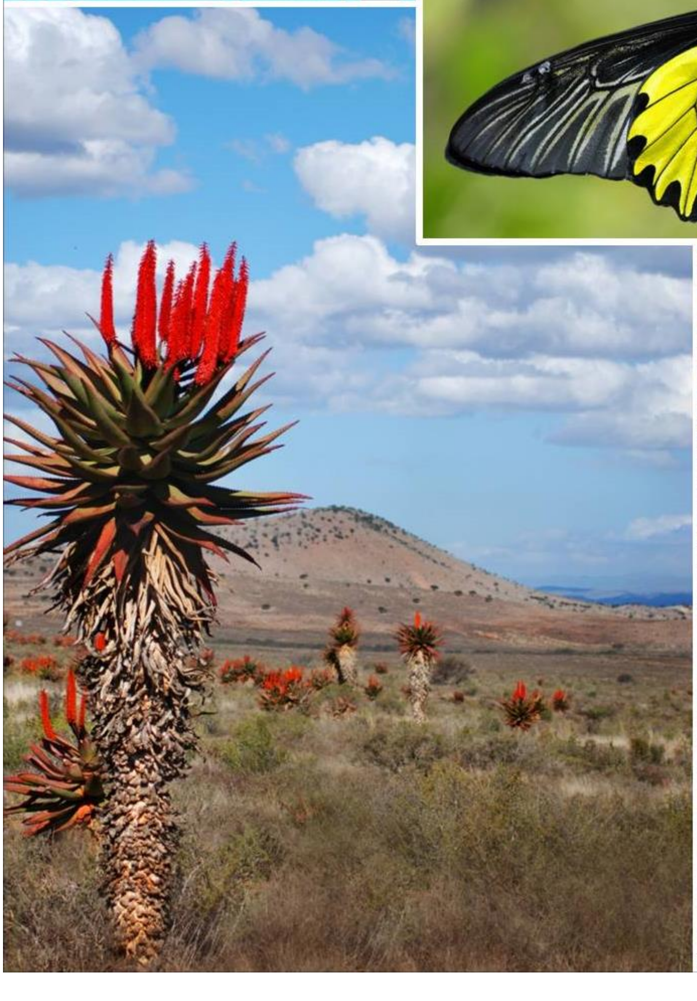
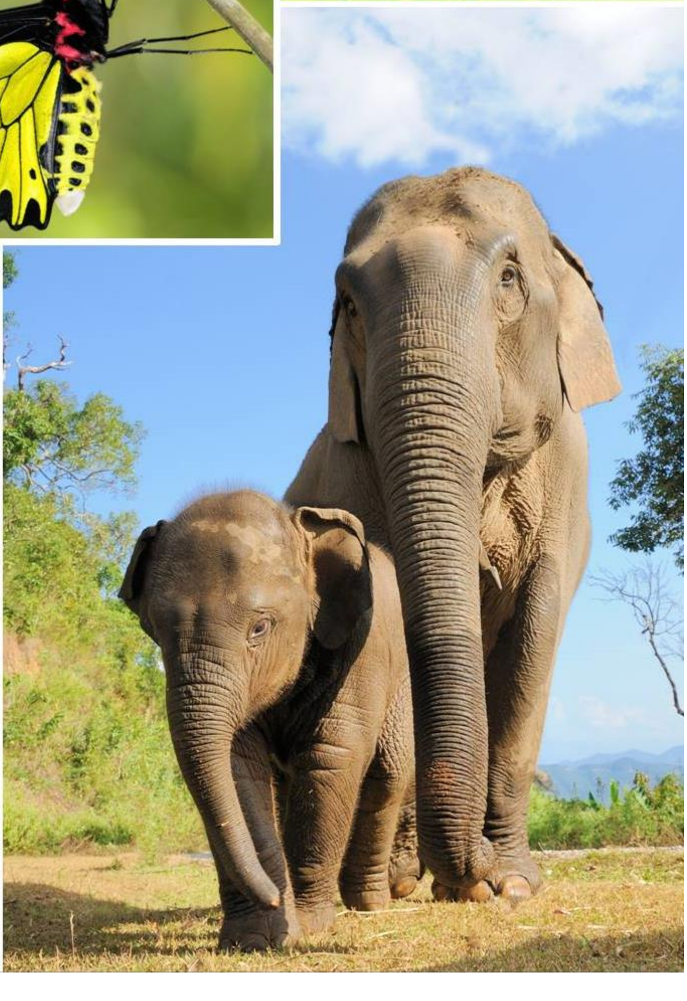

CITES Secretariat
Palais de Nations
Avenue de la Paix 8-14
CH 1211 Geneva 10
Switzerland

Tel: + 41 (0) 22 917 81 39/40
Email: info@cites.org
Website: www.cites.org

UNEP World Conservation Monitoring Centre
219 Huntingdon Road
Cambridge
CB3 0DL
United Kingdom

Tel: +44 (0) 1223 277314
Email: species@unep-wcmc.org
Website: www.unep-wcmc.org

Copyright: 2021 CITES Secretariat / United Nations Environment Programme

CITES (the Convention on International Trade in Endangered Species of Wild Fauna and Flora) is an international agreement between governments. Its aim is to ensure that international trade in specimens of wild animals and plants does not threaten their survival. At the time of writing the present introduction (March 2021), 183 States or regional economic integration organizations are party to CITES.

The UN Environment Programme World Conservation Monitoring Centre (UNEP-WCMC) is a global Centre of excellence on biodiversity. The Centre operates as a collaboration between the UN Environment Programme and the UK-registered charity WCMC. Together we are confronting the global crisis facing nature.

This publication may be reproduced for educational or non-profit purposes without special permission, provided acknowledgement to the source is made. Reuse of any figures is subject to permission from the original rights holders. No use of this publication may be made for resale or any other commercial purpose without permission in writing from the CITES Secretariat. Applications for permission, with a statement of purpose and extent of reproduction, should be sent to the CITES Secretariat, Palais des Nations, Avenue de la Paix 8-14, Geneva 10, Switzerland.

The geographical designations employed in this publication do not imply the expression of any opinion whatsoever on the part of the compilers, the CITES Secretariat or the United Nations Environment Programme concerning the legal status of any country, territory or area, or concerning the delimitation of its frontiers or boundaries.

Citation: UNEP-WCMC (Comps.) 2021. *Checklist of CITES species*. CITES Secretariat, Geneva, Switzerland and UNEP-WCMC, Cambridge, United Kingdom. Accessed on [Date].

CITES Checklist website: http://checklist.cites.org/

With financial support from: CITES Secretariat, UNEP-WCMC and the European Commission

Front cover image copyrights: Green iguana: FikMik; Blue branching hard coral: Tyler Fox; Macaw Plumage: Eduardo Rivero; Frog shadow: Patryk Kosmider; Golden Birdwing butterfly: Jason S; Aloe ferox: Tish1; and Asian elephants: Ekkachai. All cover images used under licence from Shutterstock.com

Foreword and acknowledgements

The *Checklist of CITES Species* acts as the official digest of CITES-listed species. It contains the scientific names of listed taxa according to the standard references as recognized in Resolution Conf. 12.11 (Rev. CoP18) on *Standard nomenclature*, and is regularly updated to reflect the latest information. In order to be able to implement the Convention effectively, CITES Parties need to have access to authoritative information on which species are listed, where they occur and what level of protection they are afforded. The online *Checklist* is designed to allow Parties as well as the wider public to do this, offering the opportunity to access, download and filter data on species listings, nomenclature and distributions tailored to their needs. Users can download information contained in the Checklist in the three languages of the Convention (English, French and Spanish), as well as in different formats compatible with databases and other datasets, making it easy to ensure consistency between national checklists and the nomenclature adopted by the Conference of the Parties to CITES.

The *Checklist* also offers a mechanism to allow Parties to update their own national systems with amendments to the CITES Appendices as they are made, thus avoiding duplication of effort and reducing the risk of introducing errors in copying species names. This is done via Application Programming Interface (API), which also allows Parties to "pull" the names of species and the Appendices they are included in directly into a CITES electronic permit or certificate. Information on using these web services is available from the online Checklist (and at: http://api.speciesplus.net/).

The production and publication of the *Checklist* have been made possible through the generous funding of the European Union. I would therefore like to express my sincere thanks to the European Union, not only for this project but also for their continued general support for CITES activities.

The *Checklist* is widely used and appreciated by CITES Management Authorities, Scientific Authorities, Customs officers and others around the world involved in the implementation and enforcement of CITES, as well as by intergovernmental entities, international and national non-governmental organizations, academics, the media and many others. We trust that this resource will be of value to you, and we very much welcome any feedback and suggestions on ways to improve this resource in the future.

Ivonne Higuero
CITES Secretary-General

INTRODUCTION

The Conference of the Parties recognizes the *Checklist of CITES species* as an official digest of scientific names contained in the official standard references. The *Checklist of CITES species* is now dynamically linked to Species+, a database of information on MEA-listed species that is managed by UNEP-WCMC, allowing, for the first time, taxonomic and listing changes to be reflected within this document as they are updated. This will include amendments to CITES Appendix III made between meetings of the Conference of the Parties. For this reason, it will be important for users of the *Checklist* to take note of the date of download, as outputs will change over time on the basis of changes adopted by Parties.

The Checklist website also provides the flexibility to create tailored outputs by higher taxonomic group, Appendix and country or region, with additional "Advanced options" for including or excluding elements such as Authors' names, scientific synonyms and common names. If filters have been applied, some of the descriptions below may not be applicable. For instance, if scientific synonyms have been excluded, the synonym records will not appear in the output.

Structure

The *Checklist of CITES species* comprises two parts: the *Index of CITES species* (the present output) and the *History of CITES listings*. While users can choose to download tailored outputs of the *Index of CITES species,* the complete publication is an alphabetical list of all animal and plant taxa included in the CITES Appendices. The only exceptions to this are Appendix-II orchids. These are only included if they are listed in the *CITES Orchid Checklist*, volumes 1-4, published by the Royal Botanic Gardens, Kew, or in the *Checklist for* Bulbophyllum *and allied taxa* (Sieder, Rainer & Kiehn, 2007). For the orchid species not found in these references, the *CITES Species Index* contains a record of the genera to which they belong, e.g. *Aa* spp, but not of the individual species in each genus.

Three types of record are included in the *Index of CITES species*:

1) scientific names, which are the main records and contain all information;
2) common names, which refer to the main record; and
3) scientific synonyms, which refer to the main record.

1 The scientific name record

This record is the main record that contains all the information available for each taxon.

Taxonomic names that have been officially adopted by the Conference of the Parties [see the list of standard nomenclatural references in Resolution Conf. 12.11 (Rev. CoP18) appear in boldface in the *Checklist*, e.g. ***Acinonyx jubatus*** (the cheetah). This is the case for most taxa. In some cases, nevertheless, a standard nomenclatural reference for the constituent species of a higher taxon has not yet been adopted. In such instances, a reference identified by UNEP-WCMC has been used. The names of these taxa are not in boldface, e.g. *Tridacna maxima* (or any Tridacnidae species).

Higher taxa are only included when there is an Appendix listing at that level or if the higher taxon has inherited a listing from another higher taxon. For instance, a genus within Scleractinia spp. would be included so that it would be clear to readers that all species within the genus are listed (e.g. *Acropora* spp.). If, alternatively, the inclusion in the Appendices goes no further up than the species level, as in

the case of *Dugong dugon,* the genus, family or order would not appear in this case (e.g. *Dugong,* Dugongidae and Sirenia would not appear).

Examples of scientific name records and explanation

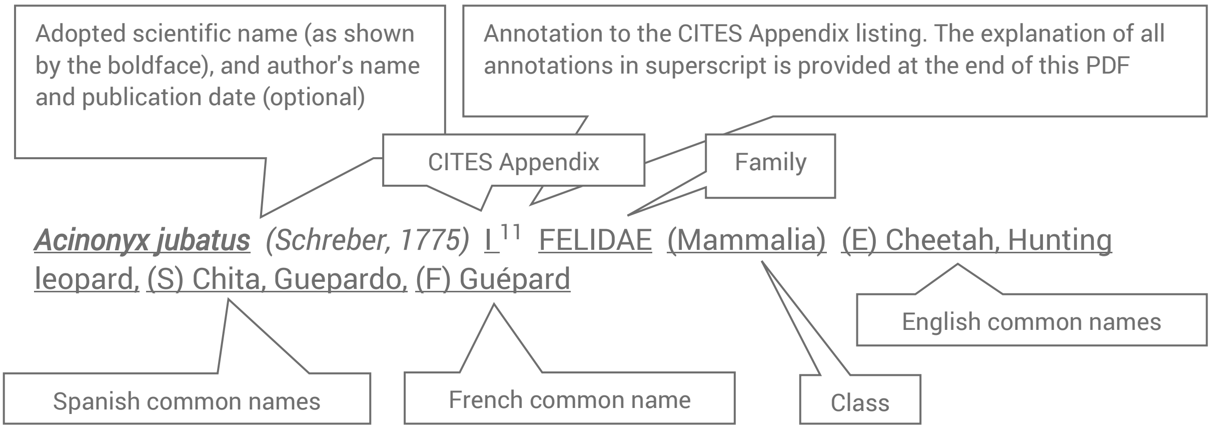

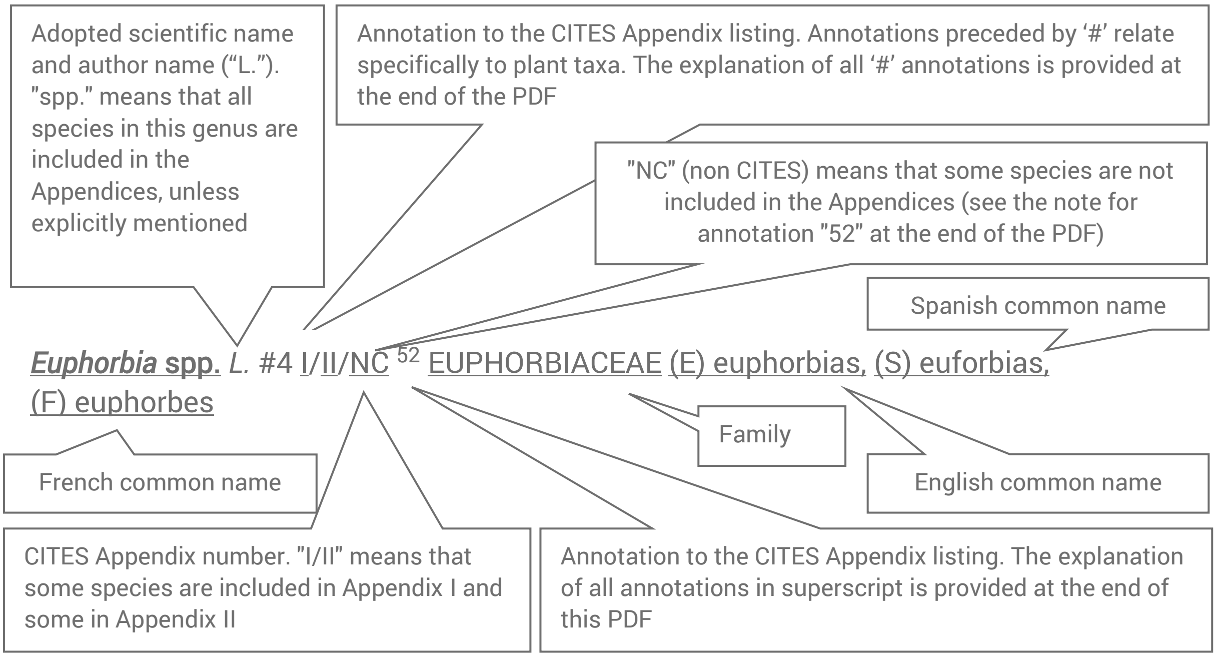

Key to abbreviations and annotations

I	listed in Appendix I
II	listed in Appendix II
III	listed in Appendix III
NC	non CITES
spp.	all species of a higher taxon
var.	variety
Superscript annotations 1 to 74.	see the key for *Annotations not preceded by "#"* at the end of this PDF
#1-#14 (flora only)	see Key to # *annotations* at the end of the PDF

2 The common name record

Where available, English (E), Spanish (S) and French (F) common names are provided. The common name is followed by the corresponding scientific name under which all information is recorded. There is only one entry for each common name, e.g. there is an entry for 'Eagle, Golden' but not for 'Golden Eagle'.

Examples of common name records and explanation

1) Hummingbird, Emerald-chinned (E): *Abeillia abeillei*

   = go to "*Abeillia abeillei*" to see the full record of the "emerald-chinned hummingbird".

2) parrots (E): PSITTACIFORMES (Aves)

   = go to "PSITTACIFORMES" to see the full record of "parrots".

3) orchids, slipper (E): *Paphiopedilum* spp. / *Phragmipedium* spp.

   = go to "*Paphiopedilum* spp." and "*Phragmipedium* spp." to see the full record of "slipper orchids", because the same common name is used for both genera.

3 The synonym record

A synonym is followed by '=' and the scientific name under which all information is recorded.

Example and explanation

Loxodonta cyclotis = *Loxodonta africana*

Explanation: *Loxodonta cyclotis* is a synonym of *Loxodonta africana.* Go to "*Loxodonta africana*" to see the full record.

Note: A same species name may be displayed as both a synonym and an accepted name when it has been given by different authors to different species. Thus, the *Index of CITES species* contains the following consecutive entries:

Porites solida = *Porites astreoides*

Porites solida II PORITIDAE (Anthozoa)

Selecting "Author's name" in the *Advanced options* will display the authors' names both on screen and in the downloads, thereby clarifying these records as:

Porites solida Verrill, 1868 = *Porites astreoides* Lamarck, 1816

Porites solida (Forskål, 1775) II PORITIDAE (Anthozoa)

In other words, the Conference of the Parties to CITES has adopted *Porites astreoides*, as named by Lamarck in 1816, and *Porites solida*, as named by Forskål in 1775, as the scientific names of two

CITES-listed species. In addition, it is recognized that the species that Verrill named *Porites solida* in 1868 is the same as the one known to CITES as *Porites astreoides*.

FLORA

Callista veratrifolia (Lindl.) Kuntze = Dendrobium lineale Rolfe
Dendrobium augustae-victoriae Kraenzl. = Dendrobium lineale Rolfe
Dendrobium cogniauxianum Kraenzl. = Dendrobium lineale Rolfe
Dendrobium grantii C.T.White = Dendrobium lineale Rolfe
Dendrobium imperatrix Kraenzl. = Dendrobium lineale Rolfe
Dendrobium lineale Rolfe #4 **II** [80] ORCHIDACEAE
Dendrobium veratrifolium Lindl. = Dendrobium lineale Rolfe
Dendrobium veratroides Bakh.f. = Dendrobium lineale Rolfe

Annotations key

Annotations not preceded by ”#”

1 ***Antilocapra americana***

Only the population of Mexico is included in Appendix I. No other population is included in the Appendices.

2 ***Bos gaurus***

Excludes the domesticated form, which is referenced as *Bos frontalis*, and is not subject to the provisions of the Convention.

3 ***Bos mutus***

Excludes the domesticated form, which is referenced as *Bos grunniens*, and is not subject to the provisions of the Convention.

4 ***Bubalus arnee***

Excludes the domesticated form, which is referenced as *Bubalus bubalis* and is not subject to the provisions of the Convention.

5 ***Ovis canadensis***

Only the population of Mexico; no other population is included in the Appendices.

6 ***Ovis gmelini***

Only the population of Cyprus; no other population is included in the Appendices

7 ***Saiga borealis***

A zero export quota for wild specimens traded for commercial purposes

8 ***Saiga tatarica***

A zero export quota for wild specimens traded for commercial purposes

9 ***Vicugna vicugna***

Only the populations of Argentina (the populations of the Provinces of Jujuy, Catamarca and Salta, and the semi-captive populations of the Provinces of Jujuy, Salta, Catamarca, La Rioja and San Juan), Chile (populations of the region of Tarapacá and of the region of Arica and Parinacota), Ecuador (the whole population), Peru (the whole population) and the Plurinational State of Bolivia (the whole population); all other populations are included in Appendix I.

For the exclusive purpose of allowing international trade in fibre from vicuñas (*Vicugna vicugna*) and their derivative products, only if the fibre comes from the shearing of live vicuñas. Trade in products derived from the fibre may only take place in accordance with the following provisions:
a) Any person or entity processing vicuña fibre to manufacture cloth and garments must request authorization from the relevant authorities of the country of origin (Countries of origin: The countries where the species occurs, that is, Argentina, Bolivia, Chile, Ecuador and Peru) to use the ”vicuña country of origin” wording, mark or logo adopted by the range States of the species that are signatories to the Convention for the Conservation and Management of the Vicuña.
b) Marketed cloth or garments must be marked or identified in accordance with the following provisions:
i) For international trade in cloth made from live-sheared vicuña fibre, whether the cloth was produced within or outside of the range States of the species, the wording, mark or logo must be used so that the country of origin can be identified. The VICUÑA [COUNTRY OF ORIGIN] wording, mark or logo has the format as detailed below:

This wording, mark or logo must appear on the reverse side of the cloth. In addition, the selvages of the cloth must bear the words VICUÑA [COUNTRY OF ORIGIN].
ii) For international trade in garments made from live-sheared vicuña fibre, whether the garments were produced within or outside of the range States of the species, the wording, mark or logo indicated in paragraph b) i) must be used. This wording, mark or logo must appear on a label on the garment itself. If the garments are produced outside of the country of origin, the name of the country where the garment was produced should also be indicated, in addition to the wording, mark or logo referred to in paragraph b) i).
c) For international trade in handicraft products made from live-sheared vicuña fibre produced within the range

States of the species, the VICUÑA [COUNTRY OF ORIGIN] - ARTESANÍA wording, mark or logo must be used as detailed below:

d) If live-sheared vicuña fibre from various countries of origin is used for the production of cloth and garments, the wording, mark or logo of each of the countries of origin of the fibre must be indicated, as detailed in paragraphs b) i) and ii).
e) All other specimens shall be deemed to be specimens of species listed in Appendix I and the trade in them shall be regulated accordingly.

10 ***Moschus* spp.**

The populations of Afghanistan, Bhutan, India, Myanmar, Nepal and Pakistan are included in Appendix I. All other populations are included in Appendix II.

10 ***Moschus* spp.**

Except the populations of Afghanistan, Bhutan, India, Myanmar, Nepal and Pakistan, which are included in Appendix I.

11 **TAYASSUIDAE spp.**

Except the species included in Appendix I (*Catagonus wagneri*) and the populations of *Pecari tajacu* of Mexico and the United States of America, which are not included in the Appendices.

12 ***Canis lupus***

Except the populations of Bhutan, India, Nepal and Pakistan, which are included in Appendix I. Excludes the domesticated form and the dingo which are referenced as *Canis lupus familiaris* and *Canis lupus dingo*.

13 **FELIDAE spp.**

Included in Appendix II, except for the species included in Appendix I. Specimens of the domesticated form are not subject to the provisions of the Convention.

14 ***Acinonyx jubatus***

Included in Appendix I. Annual export quotas for live specimens and hunting trophies are granted as follows: Botswana: 5; Namibia: 150; Zimbabwe: 50. The trade in such specimens is subject to the provisions of Article III of the Convention.

15 ***Caracal caracal***

Except the Asian population, which is included in Appendix I.

16 ***Herpailurus yagouaroundi***

Only the populations of Central and North America; all other populations are included in Appendix II.

17 ***Panthera leo***

[FAMILY listing Felidae spp.]

For *Panthera leo* (African populations): a zero annual export quota is established for specimens of bones, bone pieces, bone products, claws, skeletons, skulls and teeth removed from the wild and traded for commercial purposes. Annual export quotas for trade in bones, bone pieces, bone products, claws, skeletons, skulls and teeth for commercial purposes, derived from captive breeding operations in South Africa, will be established and communicated annually to the CITES Secretariat.

17 ***Panthera leo***

Only the populations of India; all other populations are included in Appendix II.

18 ***Prionailurus bengalensis bengalensis***

Except the populations of Bangladesh, India and Thailand, which are included in Appendix I.

19 ***Prionailurus rubiginosus***

Except the population of India, which is included in Appendix I.

19 ***Prionailurus rubiginosus***

Only the population of India; all other populations are included in Appendix II.

20 ***Puma concolor***

Only the populations of Costa Rica and Panama; all other populations are included in Appendix II

21 ***Aonyx capensis microdon***

Only the populations of Cameroon and Nigeria; all other populations are included in Appendix II.

22 ***Ursus arctos***

Except the populations of Bhutan, China, Mexico and Mongolia, which are included in Appendix I.

22 ***Ursus arctos***

Only the populations of Bhutan, China, Mexico and Mongolia; all other populations are included in Appendix II.

23 **CETACEA spp.**

Included in Appendix II, except for the species included in Appendix I. A zero annual export quota has been established for live specimens from the Black Sea population of Tursiops truncatus removed from the wild and traded for primarily commercial purposes.

24 ***Balaenoptera acutorostrata***

Population of West Greenland.

25 ***Pteropus* spp.**

Except *Pteropus brunneus* and the species included in Appendix I.

26 ***Chaetophractus nationi***

Included in Appendix II. A zero annual export quota has been established. All specimens shall be deemed to be specimens of species included in Appendix I and the trade in them shall be regulated accordingly.

27 ***Equus africanus***

Excludes the domesticated form, which is referenced as *Equus asinus* and is not subject to the provisions of the Convention.

28 ***Ceratotherium simum simum***

Only the populations of Eswatini, Namibia and South Africa; all other populations are included in Appendix I. The populations of Eswatini and South Africa are included in Appendix II for the exclusive purpose of allowing international trade in live animals to appropriate and acceptable destinations and hunting trophies. The population of Namibia is included in Appendix II for the exclusive purpose of allowing international trade in live animals for *in-situ* conservation only, and only within the natural and historical range of *Ceratotherium simum* in Africa. All other specimens shall be deemed to be specimens of species included in Appendix I and the trade in them shall be regulated accordingly.

29 ***Loxodonta africana***

The populations of Botswana, Namibia, South Africa and Zimbabwe are listed in Appendix II for the exclusive purpose of allowing:

a) trade in hunting trophies for non-commercial purposes;
b) trade in live animals to appropriate and acceptable destinations, as defined in Resolution Conf. 11.20 (Rev. CoP18), for Botswana and Zimbabwe and for *in situ* conservation programmes for Namibia and South Africa;
c) trade in hides;
d) trade in hair;
e) trade in leather goods for commercial or non-commercial purposes for Botswana, Namibia and South Africa and for non-commercial purposes for Zimbabwe;
f) trade in individually marked and certified ekipas incorporated in finished jewellery for non-commercial purposes for Namibia and ivory carvings for non-commercial purposes for Zimbabwe;
g) trade in registered raw ivory (for Botswana, Namibia, South Africa and Zimbabwe, whole tusks and pieces) subject to the following:
i) only registered government-owned stocks, originating in the State (excluding seized ivory and ivory of unknown origin);
ii) only to trading partners that have been verified by the Secretariat, in consultation with the Standing Committee, to have sufficient national legislation and domestic trade controls to ensure that the imported ivory will not be re-exported and will be managed in accordance with all requirements of Resolution Conf. 10.10 (Rev. CoP18) concerning domestic manufacturing and trade;
iii) not before the Secretariat has verified the prospective importing countries and the registered government-owned stocks;
iv) raw ivory pursuant to the conditional sale of registered government-owned ivory stocks agreed at CoP12, which are 20,000 kg (Botswana), 10,000 kg (Namibia) and 30,000 kg (South Africa);
v) in addition to the quantities agreed at CoP12, government-owned ivory from Botswana, Namibia, South Africa and Zimbabwe registered by 31 January 2007 and verified by the Secretariat may be traded and despatched, with

the ivory in paragraph g) iv) above, in a single sale per destination under strict supervision of the Secretariat;
vi) the proceeds of the trade are used exclusively for elephant conservation and community conservation and development programmes within or adjacent to the elephant range; and
vii) the additional quantities specified in paragraph g) v) above shall be traded only after the Standing Committee has agreed that the above conditions have been met; and
h) no further proposals to allow trade in elephant ivory from populations already in Appendix II shall be submitted to the Conference of the Parties for the period from CoP14 and ending nine years from the date of the single sale of ivory that is to take place in accordance with provisions in paragraphs g) i), g) ii), g) iii), g) vi) and g) vii). In addition such further proposals shall be dealt with in accordance with Decisions 16.55 and 14.78 (Rev. CoP16).

On a proposal from the Secretariat, the Standing Committee can decide to cause this trade to cease partially or completely in the event of non-compliance by exporting or importing countries, or in the case of proven detrimental impacts of the trade on other elephant populations.

All other specimens shall be deemed to be specimens of species included in Appendix I and the trade in them shall be regulated accordingly.

30 ***Chinchilla* spp.**

Specimens of the domesticated form are not subject to the provisions of the Convention

31 **FALCONIFORMES spp.**

Except *Caracara lutosa* and the species of the family Cathartidae, which are not included in the Appendices; and the species included in Appendices I and III.

32 ***Falco newtoni***

Only the population of Seychelles.

33 ***Pycnonotus zeylanicus***

Listing adopted at CoP19 in November 2022 with entry into effect delayed by 12 months.

34 **PSITTACIFORMES spp.**

Included in Appendix II, except for the species included in Appendix I and *Agapornis roseicollis, Melopsittacus undulatus, Nymphicus hollandicus* and *Psittacula krameri*, which are not included in the Appendices.

35 **STRIGIFORMES spp.**

Except *Sceloglaux albifacies* and the species included in Appendix I.

36 ***Struthio camelus***

Only the populations of Algeria, Burkina Faso, Cameroon, the Central African Republic, Chad, Mali, Mauritania, Morocco, Niger, Nigeria, Senegal and Sudan are included in Appendix I. No other population is included in the Appendices.

37 ***Caiman latirostris***

Except the population of Argentina, which is included in Appendix II, and the population of Brazil, which is included in Appendix II subject to a zero annual export quota for wild specimens traded for commercial purposes.

37 ***Caiman latirostris***

Population of Argentina, included in CROCODYLIA spp., and population of Brazil, included in CROCODYLIA spp. and subject to a zero annual export quota for wild specimens traded for commercial purposes.

38 ***Crocodylus acutus***

Population of the Integrated Management District of Mangroves of the Bay of Cispata, Tinajones, La Balsa and Surrounding Areas, Department of Córdoba, Colombia, and the population of Cuba; and the population of Mexico, which is subject to a zero export quota for wild specimens for commercial purposes

39 ***Crocodylus moreletii***

Only the population of Belize, which is included in Appendix II with a zero quota for wild specimens traded for commercial purposes, and the population of Mexico.

40 ***Crocodylus niloticus***

Included in Appendix I, except the populations of Botswana, Egypt (subject to a zero quota for wild specimens traded for commercial purposes), Ethiopia, Kenya, Madagascar, Malawi, Mozambique, Namibia, South Africa, Uganda, the United Republic of Tanzania (subject to an annual export quota of no more than 1,600 wild specimens including hunting trophies, in addition to ranched specimens), Zambia and Zimbabwe, which are included in Appendix II

40 ***Crocodylus niloticus***

Populations of Botswana, Egypt (subject to a zero quota for wild specimens traded for commercial purposes), Ethiopia, Kenya, Madagascar, Malawi, Mozambique, Namibia, South Africa, Uganda, the United Republic of Tanzania (subject to an annual export quota of no more than 1,600 wild specimens including hunting trophies, in addition to ranched specimens), Zambia and Zimbabwe.

41 ***Crocodylus porosus***

Except the populations of Australia, Indonesia, Malaysia [wild harvest restricted to the State of Sarawak and a zero quota for wild specimens for the other States of Malaysia (Sabah and Peninsular Malaysia), with no change in the zero quota unless approved by the Parties], Papua New Guinea and the Philippines [population of the Palawan Islands only, subject to a zero annual export quota for wild specimens traded for commercial purposes], which are included in Appendix II.

42 ***Ceratophora aspera***

Zero export quota for wild specimens for commercial purposes

43 ***Ceratophora stoddartii***

Zero export quota for wild specimens for commercial purposes

44 ***Lyriocephalus scutatus***

Zero export quota for wild specimens for commercial purposes

45 ***Abronia* spp.**

Except the species included in Appendix I. Zero export quota for wild specimens for *Abronia aurita, A. gaiophantasma, A. montecristoi, A. salvadorensis* and *A. vasconcelosii*.

46 **LANTHANOTIDAE spp.**

Zero export quota for wild specimens for commercial purposes.

47 ***Vipera ursinii***

Only the population of Europe, except the area which formerly constituted the Union of Soviet Socialist Republics; these latter populations are not included in the Appendices.

48 ***Chelodina mccordi***

Zero export quota for specimens from the wild.

49 ***Chelus fimbriatus***

50 ***Batagur borneoensis***

Zero quota for wild specimens for commercial purposes.

51 ***Batagur trivittata***

Zero quota for wild specimens for commercial purposes.

52 ***Heosemys annandalii***

Zero quota for wild specimens for commercial purposes.

53 ***Heosemys depressa***

Zero quota for wild specimens for commercial purposes.

54 ***Orlitia borneensis***

Zero quota for wild specimens for commercial purposes.

55 **TESTUDINIDAE spp.**

Included in Appendix II, except for the species included in Appendix I. A zero annual export quota has been established for *Centrochelys sulcata* for specimens removed from the wild and traded for primarily commercial purposes.

56 ***Agalychnis* spp.**

Includes *Agalychnis annae*, *A. callidryas*, *A. lemur* [subject to a zero annual export quota for wild-taken specimens traded for commercial purposes], *A. moreletti*, *A. saltator*, *A. spurrelli* and *A. terranova*.

57 ***Rheobatrachus* spp.**

Except *Rheobatrachus silus* and *Rheobatrachus vitellinus.*

58 ***Laotriton laoensis***

Included in Appendix II subject to a zero annual export quota for wild-taken specimens traded for commercial purposes.

59 ***Hypancistrus zebra***

Included in Appendix II subject to a zero export quota for wild specimens for commercial purposes.

60 ***Holothuria fuscogilva***

Listing adopted at CoP18 in August 2019 with entry into effect delayed by 12 months.

61 ***Holothuria nobilis***

Listing adopted at CoP18 in August 2019 with entry into effect delayed by 12 months.

62 ***Holothuria whitmaei***

Listing adopted at CoP18 in August 2019 with entry into effect delayed by 12 months.

63 ***Caribena versicolor***

Listed by the European Union

64 ***Papilio phorbanta***

Listed by the European Union

65 ***Panax ginseng***

Only the population of the Russian Federation; no other population is included in the Appendices.

66 **CACTACEAE spp.**

Except the species included in Appendix I and except *Pereskia* spp., *Pereskiopsis* spp. and *Quiabentia* spp. Artificially propagated specimens of the following hybrids and/or cultivars are not subject to the provisions of the Convention: *Hatiora* x *graeseri*; *Schlumbergera* x *buckleyi*; *Schlumbergera russelliana* x *Schlumbergera truncata*; *Schlumbergera orssichiana* x *Schlumbergera truncata*; *Schlumbergera opuntioides* x *Schlumbergera truncata*; *Schlumbergera truncata* (cultivars); Cactaceae spp. colour mutants grafted on the following grafting stocks *Harrisia* 'Jusbertii', *Hylocereus trigonus* or *Hylocereus undatus*; *Opuntia microdasys* (cultivars).

67 ***Dicksonia* spp.**

Only the populations of the Americas; no other population is included in the Appendices

68 ***Diospyros* spp.**

Populations of Madagascar.

69 ***Euphorbia* spp.**

Succulent species only except *Euphorbia misera* and the species included in Appendix I.
Artificially propagated specimens of cultivars of *Euphorbia trigona*, artificially propagated specimens of crested, fanshaped or colour mutants of *Euphorbia lactea*, when grafted on artificially propagated root stock of *Euphorbia neriifolia*, and artificially propagated specimens of cultivars of *Euphorbia* 'Milii' when they are traded in shipments of 100 or more plants and readily recognizable as artificially propagated specimens, are not subject to the provisions of the Convention.

70 ***Euphorbia cremersii***

Included in Appendix I. Includes the forma *viridifolia* and the variety *rakotozafyi*.

71 ***Euphorbia cylindrifolia***

Included in Appendix I. Includes the subspecies *tuberifera*.

72 ***Euphorbia decaryi***

Included in Appendix I. Includes the varieties *ampanihyensis, robinsonii and spirosticha*.

73 ***Euphorbia moratii***

Included in Appendix I. Includes the varieties *antsingiensis, bemarahensis* and *multiflora*.

74 ***Aloe* spp.**

Except the species included in Appendix I. Also excludes *Aloe vera*, also referenced as *Aloe barbadensis* which is not included in the Appendices.

75 ***Aloe compressa***

Included in Appendix I. Includes the varieties *paucituberculata, rugosquamosa* and *schistophila*.

76 ***Aloe haworthioides***

Included in Appendix I. Includes the variety *aurantiaca*.

77 ***Aloe laeta***

Included in Appendix I. Includes the variety *maniaensis*.

78 ***Cedrela* spp.**

Populations of the Neotropics.
Listing adopted at CoP18 in August 2019 with entry into effect delayed by 12 months.

79 ***Swietenia macrophylla***

Populations of the Neotropics

80 **ORCHIDACEAE spp.**

Included in Appendix II, except for the species included in Appendix I.
Additionally, artificially propagated hybrids of the following genera are not subject to the provisions of the Convention, if conditions, as indicated under a) and b), are met: *Cymbidium, Dendrobium, Phalaenopsis* and *Vanda*:
a) Specimens are readily recognizable as artificially propagated and do not show any signs of having been collected in the wild such as mechanical damage or strong dehydration resulting from collection, irregular growth and heterogeneous size and shape within a taxon and shipment, algae or other epiphyllous organisms adhering to leaves, or damage by insects or other pests; and
b) i) when shipped in non-flowering state, the specimens must be traded in shipments consisting of individual containers (such as cartons, boxes, crates or individual shelves of CC-containers) each containing 20 or more plants of the same hybrid; the plants within each container must exhibit a high degree of uniformity and healthiness; and the shipment must be accompanied by documentation, such as an invoice, which clearly states the number of plants of each hybrid; or
ii) when shipped in flowering state, with at least one fully open flower per specimen, no minimum number of specimens per shipment is required but specimens must be professionally processed for commercial retail sale, e.g. labelled with printed labels or packaged with printed packages indicating the name of the hybrid and the country of final processing. This should be clearly visible and allow easy verification.
Plants not clearly qualifying for the exemption must be accompanied by appropriate CITES documents.

81 ***Aerangis ellisii***

Included in Appendix I. Seedling or tissue cultures obtained *in vitro* and transported in sterile containers are not subject to the provisions of the Convention only if the specimens meet the definition of 'artificially propagated' agreed by the Conference of the Parties in Resolution Conf. 11.11 (Rev. CoP18), i.e. plant specimens: a) grown under controlled conditions; and b) grown from seeds, cuttings, divisions, callus tissues or other plant tissues, spores or other propagules that either are exempt from the provisions of the Convention or have been derived from cultivated parental stock.

82 ***Cattleya jongheana***

Included in Appendix I. Seedling or tissue cultures obtained *in vitro* and transported in sterile containers are not subject to the provisions of the Convention only if the specimens meet the definition of 'artificially propagated' agreed by the Conference of the Parties in Resolution Conf. 11.11 (Rev. CoP18), i.e. plant specimens: a) grown under controlled conditions; and b) grown from seeds, cuttings, divisions, callus tissues or other plant tissues, spores or other propagules that either are exempt from the provisions of the Convention or have been derived from cultivated parental stock.

83 ***Cattleya lobata***

Included in Appendix I. Seedling or tissue cultures obtained *in vitro* and transported in sterile containers are not subject to the provisions of the Convention only if the specimens meet the definition of 'artificially propagated' agreed by the Conference of the Parties in Resolution Conf. 11.11 (Rev. CoP18), i.e. plant specimens: a) grown under controlled conditions; and b) grown from seeds, cuttings, divisions, callus tissues or other plant tissues, spores or other propagules that either are exempt from the provisions of the Convention or have been derived from cultivated parental stock.

84 ***Dendrobium cruentum***

Included in Appendix I. Seedling or tissue cultures obtained *in vitro* and transported in sterile containers are not subject to the provisions of the Convention only if the specimens meet the definition of 'artificially propagated' agreed by the Conference of the Parties in Resolution Conf. 11.11 (Rev. CoP18), i.e. plant specimens: a) grown under controlled conditions; and b) grown from seeds, cuttings, divisions, callus tissues or other plant tissues, spores or other propagules that either are exempt from the provisions of the Convention or have been derived from cultivated parental stock.

85 ***Mexipedium xerophyticum***

Included in Appendix I. Seedling or tissue cultures obtained *in vitro* and transported in sterile containers are not subject to the provisions of the Convention only if the specimens meet the definition of 'artificially propagated' agreed by the Conference of the Parties in Resolution Conf. 11.11 (Rev. CoP18), i.e. plant specimens: a) grown under controlled conditions; and b) grown from seeds, cuttings, divisions, callus tissues or other plant tissues,

spores or other propagules that either are exempt from the provisions of the Convention or have been derived from cultivated parental stock.

86 ***Paphiopedilum* spp.**

Included in Appendix I. Seedling or tissue cultures obtained *in vitro* and transported in sterile containers are not subject to the provisions of the Convention only if the specimens meet the definition of ‘artificially propagated’ agreed by the Conference of the Parties in Resolution Conf. 11.11 (Rev. CoP18), i.e. plant specimens: a) grown under controlled conditions; and b) grown from seeds, cuttings, divisions, callus tissues or other plant tissues, spores or other propagules that either are exempt from the provisions of the Convention or have been derived from cultivated parental stock.

87 ***Peristeria elata***

Included in Appendix I. Seedling or tissue cultures obtained *in vitro* and transported in sterile containers are not subject to the provisions of the Convention only if the specimens meet the definition of ‘artificially propagated’ agreed by the Conference of the Parties in Resolution Conf. 11.11 (Rev. CoP18), i.e. plant specimens: a) grown under controlled conditions; and b) grown from seeds, cuttings, divisions, callus tissues or other plant tissues, spores or other propagules that either are exempt from the provisions of the Convention or have been derived from cultivated parental stock.

88 ***Phragmipedium* spp.**

Included in Appendix I. Seedling or tissue cultures obtained *in vitro* and transported in sterile containers are not subject to the provisions of the Convention only if the specimens meet the definition of ‘artificially propagated’ agreed by the Conference of the Parties in Resolution Conf. 11.11 (Rev. CoP18), i.e. plant specimens: a) grown under controlled conditions; and b) grown from seeds, cuttings, divisions, callus tissues or other plant tissues, spores or other propagules that either are exempt from the provisions of the Convention or have been derived from cultivated parental stock.

89 ***Renanthera imschootiana***

Included in Appendix I. Seedling or tissue cultures obtained *in vitro* and transported in sterile containers are not subject to the provisions of the Convention only if the specimens meet the definition of ‘artificially propagated’ agreed by the Conference of the Parties in Resolution Conf. 11.11 (Rev. CoP18), i.e. plant specimens: a) grown under controlled conditions; and b) grown from seeds, cuttings, divisions, callus tissues or other plant tissues, spores or other propagules that either are exempt from the provisions of the Convention or have been derived from cultivated parental stock.

90 ***Cyclamen* spp.**

Artificially propagated specimens of cultivars of *Cyclamen persicum* are not subject to the provisions of the Convention. However, the exemption does not apply to such specimens traded as dormant tubers.

91 ***Osyris lanceolata***

Populations of Burundi, Ethiopia, Kenya, Rwanda, Uganda and the United Republic of Tanzania.

92 ***Picrorhiza kurrooa***

Excludes *Picrorhiza scrophulariiflora*.

93 ***Taxus chinensis***

Includes infraspecific taxa of this species

94 ***Taxus cuspidata***

Includes infraspecific taxa of this species. Artificially propagated hybrids and cultivars of *Taxus cuspidata*, live, in pots or other small containers, each consignment being accompanied by a label or document stating the name of the taxon or taxa and the text ”artificially propagated”, are not subject to the provisions of the Convention.

95 ***Taxus fuana***

Includes infraspecific taxa of this species.

96 ***Taxus sumatrana***

Includes infraspecific taxa of this species.

97 ***Siphonochilus aethiopicus***

Populations of Mozambique, South Africa, Eswatini and Zimbabwe.

Annotations preceded by ”#”

Annotations are used in the CITES Appendices to indicate which population, parts or derivatives are concerned by the listing or to clarify its scope. The meaning of the # annotations (applicable to flora only) has changed over the years. The # annotations that are currently valid are those adopted at the 16th Conference of the Parties (CoP 16). These are provided below.

CoP19	Valid from 23/02/2023
#1	All parts and derivatives, except:<br>a) seeds, spores and pollen (including pollinia);<br>b) seedling or tissue cultures obtained *in vitro* transported in sterile containers;<br>c) cut flowers of artificially propagated plants; and<br>d) fruits, and parts and derivatives thereof, of artificially propagated plants of the genus *Vanilla*
#2	All parts and derivatives except:<br>a) seeds and pollen; and<br>b) finished products packaged and ready for retail trade.
#3	Whole and sliced roots and parts of roots, excluding manufactured parts or derivatives, such as powders, pills, extracts, tonics, teas and confectionery.
#4	All parts and derivatives, except:<br>a) seeds (including seedpods of Orchidaceae), spores and pollen (including pollinia). The exemption does not apply to seeds from Cactaceae spp. exported from Mexico, and to seeds from *Beccariophoenix madagascariensis* and *Dypsis decaryi* exported from Madagascar;<br>b) seedling or tissue cultures obtained *in vitro* transported in sterile containers;<br>c) cut flowers of artificially propagated plants;<br>d) fruits, and parts and derivatives thereof, of naturalized or artificially propagated plants of the genus *Vanilla* (Orchidaceae) and of the family Cactaceae;<br>e) stems, flowers, and parts and derivatives thereof, of naturalized or artificially propagated plants of the genera *Opuntia* subgenus *Opuntia* and *Selenicereus* (Cactaceae);<br>f) finished products of *Aloe ferox* and *Euphorbia antisyphilitica* packaged and ready for retail trade; and<br>g) finished products derived from artificial propagation, packaged and ready for retail trade of cosmetics containing parts and derivatives of *Bletilla striata*, *Cycnoches cooperi*, *Gastrodia elata*, *Phalaenopsis amabilis* or *Phalaenopsis lobbii*.
#5	Logs, sawn wood and veneer sheets.
#6	Logs, sawn wood, veneer sheets and plywood.
#7	Logs, woodchips, powder and extracts.
#8	Underground parts (i.e. roots, rhizomes): whole, parts and powdered.
#9	All parts and derivatives except those bearing a label:<br>“Produced from *Hoodia* spp. material obtained through controlled harvesting and production under the terms of an agreement with the relevant CITES Management Authority of [Botswana under agreement No. BW/xxxxxx] [Namibia under agreement No. NA/xxxxxx] [South Africa under agreement No. ZA/xxxxxx]”.
#10	All parts, derivatives and finished products, except re-export of finished musical instruments, finished musical instrument accessories and finished musical instrument parts.
#11	Logs, sawn wood, veneer sheets, plywood, powder and extracts. Finished products containing such extracts as ingredients, including fragrances, are not considered to be covered by this annotation.
#12	Logs, sawn wood, veneer sheets, plywood and extracts. Finished products containing such extracts as ingredients, including fragrances, are not considered to be covered by this annotation.
#13	The kernel (also known as ’endosperm’, ’pulp’ or ’copra’) and any derivatives thereof, except finished products packaged and ready for retail trade.
#14	All parts and derivatives except:<br>a) seeds and pollen;<br>b) seedling or tissue cultures obtained *in vitro* transported in sterile containers;<br>c) fruits;<br>d) leaves;<br>e) exhausted agarwood powder, including compressed powder in all shapes; and<br>f) finished products packaged and ready for retail trade, this exemption does not apply to wood chips, beads, prayer beads and carvings.

#15 All parts and derivatives, except:
a) Leaves, flowers, pollen, fruits, and seeds;
b) Finished products to a maximum weight of wood of the listed species of up to 10 kg per shipment;
c) Finished musical instruments, finished musical instrument parts and finished musical instrument accessories;
d) Parts and derivatives of *Dalbergia cochinchinensis*, which are covered by Annotation # 4; and
e) Parts and derivatives of *Dalbergia* spp. originating and exported from Mexico, which are covered by Annotation # 6.

#16 Seeds, fruits and oils.

#17 Logs, sawn wood, veneer sheets, plywood and transformed wood.

#18 Excluding parts and derivatives, other than eggs
